# Work-Related Triggers of Mental Illness Relapse in South African Teachers

**DOI:** 10.3390/ijerph22060923

**Published:** 2025-06-11

**Authors:** Thembi Nkomo, Mokoko Percy Kekana, Mabitsela Hezekiel Mphasha

**Affiliations:** Department of Public Health, University of Limpopo, R71 Tzaneen Road and University Street, Polokwane 0727, South Africa

**Keywords:** mental illness, mental illness relapse, work-related triggers of mental illness relapse, occupational health, health and well-being

## Abstract

Teachers with mental illness are vulnerable to work-related triggers that can lead to relapse, affecting their mental health and ability to recover, stay employed, and deliver quality education. This empirical study explored such triggers among public school teachers in Limpopo Province, South Africa. Using Bronfenbrenner’s Ecological Systems Theory, a qualitative phenomenological design was adopted. Semi-structured face-to-face interviews were conducted with 14 participants that were purposively selected across four hospitals. Data were audio-recorded, transcribed verbatim, and analyzed using Tesch’s eight-step open-coding method. Findings revealed being gossiped about by colleagues, excessive workload, limited leadership and parental support, classroom management challenges, high performance expectations without support, and inadequate teacher mental health policies in schools. These triggers can lead to frequent absenteeism and poor teaching outcomes. They will further increase the risk of medication resistance and long-term cognitive decline due to progressive structural brain damage as a result of multiple relapses. The study highlights the urgent need for multi-stakeholder collaboration, including clinicians, academic institutions, union representatives, and the Department of Basic Education, to co-develop effective, context-sensitive strategies to mitigate work-related triggers of mental illness relapse. These strategies are not only essential for enabling long-term workforce participation but also advancing sustainable mental health and well-being.

## 1. Introduction

Mental illness is a chronic condition of the mind that affects one’s cognitive and emotional functions and encompasses a broad spectrum of disorders like depression, schizophrenia, anxiety, and bipolar disorder [1]. Workers with mental illness face challenges regarding job retention, potential for discrimination, and impaired quality of life [2]. These challenges are more prominent in certain professions such as teachers, where the teaching profession is recognized for its high levels of stress. Teachers play a vital role in shaping future generations [3], however, the unique demands of teaching, including heavy workloads, and emotional labor can serve as a trigger and make them more vulnerable to mental illness relapse [4]. Relapse in mental illness occurs when an individual returns to the acute phase of their mental illness condition. This is when a person starts to see or hear things that do not exist or behaves in a disorganized manner after partial recovery [3]. This may consequently result in hospitalization, treatment resistance, and cognitive impairment due to progressive structural brain damage if not addressed [5]. Globally, the relapse rate of mental illness varies from moderate rates of 50% to severe rates of 92% [6]. In 2020, in Japan, the prevalence rate of mental illness relapse among public school teachers was at 60% [7]. In Nigeria, it was found to be 67.1% [8] and 61.8% in South Africa [9]. Various studies have been conducted on the work-related aspects that affect teachers’ mental health. In the United Kingdom, Thompson [10] found that teachers were under pressure to ensure that learners get good grades. A study conducted in the Philippines found that teachers experience an elevated level of workload where new demands were added without removing other work tasks [11]. They concluded the study by suggesting that school authorities systematically provide a balanced workload for teachers and review the workload of teachers as a move towards improving the quality of teaching in public schools [11]. In Kenya, it was observed that teachers perceived their workload as substantial, exacerbated by limited resources, and they concluded the study by recommending the employment of more teachers to lessen the workload challenges [12]. A study conducted in South Africa noted that teachers reported stress from curriculum changes due to insufficient training on implementation [13]. The study concluded with a recommendation for enhanced support interventions at both school and departmental levels to prevent burnout. While the above studies have made contributions to work-related aspects that affect teachers’ mental health, there are insufficient studies focusing on teachers with existing mental illness, highlighting a gap in the research on work-related triggers of mental illness relapse among teachers. In light of this, the purpose of the study was to explore work-related triggers of mental illness relapse among public school teachers within the South African context, with a research question: What are the work-related triggers of mental illness relapse among public school teachers in Limpopo Province? This study is part of the principal investigator’s ongoing PhD project, which aims to develop and validate a strategy to minimize mental illness relapse among public school teachers in Limpopo Province. Limpopo Province was selected because it has the highest number of dysfunctional schools in South Africa [14]. Dysfunctional schools are typically characterized by poor, weak leadership and low learner performance [15]. These challenging conditions negatively affect overall school performance and place additional stress on teachers, contributing to teachers’ poor psychological well-being [16]. This study used an exploratory qualitative research approach, guided by Bronfenbrenner’s Ecological Systems Theory [17]. Participants were purposively selected, and this amounted to 14 participants, which were determined by data saturation. All participants were public school teachers working in both primary and high school, with mental illness and a history of mental illness relapse of more than once. Data were collected through semi-structured in-depth interviews and analyzed using Tesch’s eight-step open coding method. Understanding the work-related triggers of mental illness relapse is essential to developing targeted interventions that can improve mental health outcomes for teachers with mental illness and ensure sustainable work productivity and health and well-being in the workplace of teachers with mental illness. By addressing these triggers, teachers can be supported in maintaining their recovery and preventing further mental illness relapse, thereby enabling them to stay at work longer and be healthy in the workplace.

### Theoretical Framework

This study was guided by the Bronfenbrenner’s Ecological Systems Theory developed by Bronfenbrenner in 1977 [17], which examines how individuals are influenced by various systems. The strength of this theory is that it acknowledges that there is an interplay between different human, relational, group, and societal influences. This, therefore, makes the theory applicable and relevant in understanding the work-related triggers of mental illness relapse among public school teachers because the underlying work-related triggers may originate from multiple work-related factors within the school environment and not solely from their teaching duties. The theory identifies five interrelated systems (microsystem, mesosystem, exosystem, macrosystem, and chronosystem). However, for the purpose of the study, the researchers explored three interrelated systems (microsystem, mesosystem, exosystem), as they are the most relevant to exploring the work-related triggers of mental illness relapse. The researchers used the three systems as a guide to explore how the three interrelated systems trigger the public school teachers’ mental illness relapse in the workplace and they were applied to the study in the following ways:

Microsystem: The researchers explored their immediate environment, which they directly interact with, such as workload and interactions with their learners.

Mesosystem: This system explored the interactions between the participants (teachers) and key structures within the school environment, particularly their work-related experiences with the school management team and colleagues.

Exosystem: This system explored how their indirect environment, such as how the Department of Basic Education policies, contributes to triggering their mental illness relapse. While studies from various countries, such as Australia and South Africa, made contributions to the body of knowledge using Bronfenbrenner’s Ecological Systems Theory [17], they mostly focused on examining learners’ and teachers’ mental health [18,19], leaving a gap on using this framework [17] to focus on teachers with existing mental illness and how work-related factors serve as triggers to their mental illness relapse. This study, therefore, contributes to theory development by extending the application of Bronfenbrenner’s Ecological Systems Theory [17] to the teacher’s work-related triggers of mental illness relapse phenomena, especially in low-resource settings such as the public schools in South Africa. This will assist in shaping and directing interventions that will realistically and holistically aim at addressing work-related triggers of mental illness relapse in various systems within the schools. Access to the participants (public school teachers with mental illness and a history of mental illness relapse of more than once) was facilitated through the psychiatric units of public hospitals and a private psychiatric hospital where the participants were receiving mental health services. This route was chosen to ensure purposive sampling of the participants, who were engaged in ongoing care, making them suitable for understanding and having the lived experience of work-related triggers of mental illness relapse.

## 2. Materials and Methods

### 2.1. Study Design

This study used an exploratory qualitative research approach [20]. This qualitative approach was informed by the Bronfenbrenner’s Ecological Systems Theory [17], which allowed for an in-depth exploration of the participants’ subjective experiences on the work-related triggers of mental illness relapse and this made it suitable for the study’s objective, which was to explore the work-related triggers of mental illness relapse among public school teachers in Limpopo Province, South Africa. The theory [17] guided the development of interview questions and the interpretation of findings by helping us to categorize and analyze experiences at the microsystem, mesosystem, and exosystem levels.

### 2.2. Setting and Sampling

Data were collected from four different hospitals and districts in Limpopo Province, which comprised two specialized psychiatry hospitals (one state and one private), one regional hospital, and one tertiary and academic hospital. All hospitals are part of the main hospitals in the province that provide treatment and rehabilitation for persons with mental illness, and they also serve as referral hospitals (various hospitals in the province refer to these hospitals for management and rehabilitation after stabilizing the patients), and this is the reason why they were chosen as study settings. Participants were selected in the ward and in the psychiatry outpatient department. Purposive sampling was used to select eligible participants, who were teachers with mental illness, had a history of mental illness relapse of more than once, and were stable. For the patients that were in the ward, they also had to be stable and awaiting discharge, and this resulted in 14 participants, which were determined by data saturation, and this was when there was no new information and themes being generated. Eligible participants who were not stable and those who did not wish to give consent were excluded from the study. Participants were referred to the primary investigator by the treating psychiatrists and the psychiatric nurses, as they were the ones who assessed the patients and recommended stable teachers who were eligible for the study.

### 2.3. Data Collection

Data collection started after obtaining all ethical clearance (first from the Turfloop Research Ethics committee, then the Limpopo Province Department of Health, and the Chief Executive Officers of all the hospitals where data was collected). Verbal permission to interview participants at a private psychiatric hospital was granted by their admitting doctors. All participants manually signed an informed consent form prior to their participation in the study.

Data were collected through the use of individual face-to-face in-depth semi-structured interviews; voice recorders were used to record the interviews, and field notes were taken. A pilot study was conducted at a regional hospital with two participants to test the feasibility of the interview guide [21], and no modifications to the interview guide were required. The interview guide was formulated and guided by Bronfenbrenner’s Ecological Systems Theory [17], various existing literature reviews, and consultations with experts in the field (comprising occupational therapists, psychiatrists, and the Department of Basic Education’s employee health and wellness personnel).

All interviews were conducted in English. However, some participants also used their first language (mainly Sepedi and Xitsonga) but frequently responded in English. All the interviews were conducted by the primary investigator, who is fluent in English, Sepedi, and Xitsonga. The primary investigator’s linguistic fluency significantly contributed to establishing rapport with all the participants, and this created a comfortable and culturally sensitive environment that encouraged the participants to speak openly about their work-related triggers of their mental illness relapse. The leading question posed to all participants was: What are your perceived work-related triggers of your mental illness relapse? This question was designed to explore the work-related triggers of mental illness relapse by examining the environmental factors as guided by Bronfenbrenner’s Ecological Systems Framework [17]. The main question was followed by subquestions and open-ended probing questions to elicit detailed information about the participants’ subjective experience on their work-related triggers of their mental illness relapse [22].

Probing questions were tailored to each participant’s responses to encourage elaboration and provide clarity. Moreover, clarity-seeking questions were also used to gain deeper insights, ensuring a comprehensive understanding of each participant’s experiences in relation to the work-related triggers of their mental illness relapse within their ecological context [23]. Active listening techniques, such as paraphrasing participants’ responses, nodding, and maintaining appropriate eye contact, were used to show attentiveness and foster rapport, which also encouraged the participants to express themselves in detail [24].

### 2.4. Data Analysis

Data were analyzed manually, using Tesch’s eight-step open coding method proposed by Creswell [20]. This process began with Step 1, which involved listening to voice recordings, transcribing interviews, translating some parts of the interviews from Xitsonga and Sepedi to English, thoroughly reading the verbatim transcripts to familiarize with the data, and integrating field notes into corresponding transcripts. In Step 2, two copies of the transcripts were made; one was the master copy, and the other was a copy to write the researcher’s thoughts. One transcript was selected for initial analysis, with underlying meanings and impressions noted in the margins, paragraphs, and pages numbered. Step 3 involved analyzing each transcript, clustering related topics into columns, color coding, and abbreviating topics into codes, and the initial codes were not considered final. Instead, they were revisited and revised in light of new insights gained from subsequent steps. Similarly, in Step 4, where codes were written next to text segments and checked for emerging categories, the categories were not fixed but were open to change as the analysis progressed. In Step 5, the most descriptive wording for topics was identified and turned into categories, with related topics grouped and interrelationships indicated. The categories were further defined and alphabetized in Step 6, but this was performed with an understanding that the analysis was ongoing. Step 7 involved assembling data for each category to conduct preliminary analysis and refine themes, which were then finalized. Step 8 of recoding was not performed as there was no need to recode.

### 2.5. Ethical Considerations

The researchers obtained ethics approval to conduct the study from the Turfloop Research Ethics Committee (TREC, project number: TREC/1545/2024: PG), Limpopo Department of Health (Ref: LP_2024-08-027), the tertiary and academic hospital (Ref: S5/3/1/2), the regional hospital (S5/4/2), and the specialized psychiatry hospital where the study was conducted (S5/3/1/2). Data was also collected from a private specialized psychiatry hospital, and the admitting doctors gave verbal consent for the primary investigator to interview their patients.

All participants volunteered to participate in the original study. None of the participants were bribed or forced; they all did so of their own free will. They were all informed about the research study and encouraged to feel free to quit along the way should they not feel comfortable continuing. A written informed consent form was also explained in detail, and participants were asked if they understood what it entailed before signing.

## 3. Results

Table 1 shows that 14 participants were part of the study. Six participants were male public school teachers, eight participants were female public school teachers, and they all had a history of mental illness relapse.

Table 2 indicates three themes and seven subthemes that emerged from the analyzed data, drawing on Bronfenbrenner’s Ecological Systems Theory [17]. The themes were named and organized according to the theory [17] with correlating subthemes.

The results below are based on the themes and subthemes that emerged from the data analyses. They provide a comprehensive understanding of the work-related triggers of mental illness relapse among public school teachers in Limpopo Province through the lens of Bronfenbrenner’s Ecological Systems Theory [17] and are further discussed below:

### 3.1. Theme 1: Microsystem

This theme represents the immediate environment in which the participants directly interact with one another daily, and this includes their workload and classroom factors. It explores how they are potential work-related triggers of mental illness relapse. The subthemes are further explored below:

#### 3.1.1. Subtheme 1.1: Excessive Workload and Administrative Responsibilities

Participants described the challenges of managing administrative tasks alongside their teaching duties. They mentioned that administrative tasks often exceeded their teaching responsibilities, making it difficult to keep up. The following participant quotations reflect their experiences:

“What I see as a trigger is the multiple admin work that I have to do, which is more than the teaching that I do, and this is insane because I struggle to keep up.”

(Participant 1)

“The admin work we are expected to do is much more than the teaching itself, and it is frustrating because I struggle to keep up.”

(Participant 7)

“I had plenty of marking to do because I have bigger classes, but at least now that they employ teaching assistants, I was able to ask one of them to assist me in marking. But they kept on giving her work, which delayed my marking, and this got me frustrated and in a panic mode, I had to ask my daughter to assist with the marking due to time constraints.”

(Participant 14)

Fieldnotes: Participant 14’s body language conveyed signs of stress and impatience as she was tightening her jaw when she described the initial frustration with marking and the delays caused by the teaching assistants being given other tasks. She also sounded frustrated when she spoke about the delay in marking and the need to ask their daughter for help (possibly reflecting a heightened state of anxiety or panic).

#### 3.1.2. Subtheme 1.2: Classroom Factors

Participants reported difficulty maintaining order in the classroom and having to deal with difficult learners. Their responses are further elaborated below:

“Most learners in my class are destructive; they make a lot of noise, and I struggle to maintain order in my classroom. My colleagues and the principal frequently come to my class to tell the leaners to keep quiet, afterwards they tell me that the leaners are making noise, as if I’m not frustrated already and this really affects me because I know that the day will not end without them coming to my class, this also makes me to drag my feet when I go to work.”

(Participant 4)

“I had an altercation with one of the leaners the other day. That boy disrespected me so bad, and he even told me that I would not do anything because if I touched him, he would open a police case for me. He even used vulgar words on me, I had to walk away but if it was me who did that, all hell was going to break loose, the parents would be at school in less than 10 min, and they would even report me to the MEC (Member of the Executive Counsil)and I could even get suspended but because it’s a leaner doing that to me, I had to take it in and continue with my life, as if nothing happened. I am a human being, you know, and I get affected by such things, but the policy does not allow me to defend myself, meaning that I would rather be killed by a leaner than defend myself.”

(Participant 12)

Fieldnotes: Participant 12 appeared visibly upset and angry while discussing the altercation, with a tense tone and clenched fists. His frustration was evident, and he expressed a sense of powerlessness and unfairness in the situation. There was a clear emotional impact as he voiced feelings of being dehumanized and unsupported by the policies that prevent him from defending himself.

The participants’ lived work experiences indicate the influence that their microsystem has on their health and well-being, and this is because participants expressed dragging their feet to work due to altercations they had with their leaders, and some had to rely on external help to cope with the demands of their microsystem. This highlights the need for supportive measures to manage workload and classroom issues in order to ensure that such work-related triggers of mental illness relapse emanating from the microsystem are minimized or managed.

### 3.2. Theme 2: Mesosystem

This theme yielded three subthemes. It explored the interactions between the participants (teachers) and key structures within the school environment, particularly their work-related experiences with the school management team and colleagues. The subthemes are further explored below:

#### 3.2.1. Theme 2.1 Gossip in the Workplace

Participants reported that they were gossiped about by their colleagues, which affected their confidence and also served as a trigger for their mental illness relapse, as expressed by the participants in their responses below:

“One of the colleagues told me that another colleague, whom I won’t mention, was gossiping about me and said I’m always relapsing and hardly at work…. There is a lot of gossip at work.... That environment is not a safe one for people like me because it affects my mental health and my confidence and makes me hate going to work.”

(Participant 1)

“I turned from hero to zero after disclosing my mental illness to some of my colleagues. They told other colleagues and they frequently gossiped about me at work, and this affected my self-esteem, I had multiple relapses one after the other, it was just too much for me to take in and this also affected my work because I was no longer happy at work, I started to have backlogs of the teaching content and the administrative tasks.”

(Participant 10)

#### 3.2.2. Subtheme 2.2: Disclosure of Mental Health Conditions in the Workplace Leads to Decreased Productivity

Teachers who disclosed their mental health conditions reported experiencing increased scrutiny, which they reported to have affected their self-esteem and well-being. The participants’ experiences are further elaborated below:

“Everything I do is being questioned and linked to my mental illness. I really regret the day I disclosed to the school principal and the HoD (Head of Department). This constant scrutiny has made it difficult for me to focus on my work because I feel like I have to work twice as hard to prove myself and this makes me feel demotivated to go to work, so I end up taking sick leave or leave work earlier and this affects me because my workload keeps piling up, creating a backlog that I struggle to catch up on, and ultimately affecting my performance.”

(Participant 3)

“Trust me I’m not the only one who struggles with most of the teaching content and the workload but because I have mental illness, of which I have disclosed to the school management team, I’m always the one being picked on. I find myself spending more time trying to cope with the stress and self-doubt instead of focusing on lesson preparation and teaching effectively. This not only slows my productivity but also affects the quality of my work in the classroom.”

(Participant 7)

Fieldnotes: Participant 7 appeared visibly frustrated and defensive while speaking, using hand gestures to emphasize her points about being unfairly singled out. There was also a sense of exhaustion in her voice when she described the struggle between managing her mental health and meeting the demands of her job.

#### 3.2.3. Subtheme 2.3: Workplace Exclusion and Lack of Support from Colleagues

In this subtheme, teachers reported difficulty in seeking help due to the unwelcoming attitudes of their colleagues. Participants reported that they feel like a burden when asking for assistance. The participants’ experiences are further outlined in the following quotes:

“I feel like I’m a burden because I’m always consulting and asking questions, some are helpful, but most are rude, they will just say they don’t know, but they have been working there for more than 10 years. It causes me anxiety because I’m fairly new, I feel like I’m still finding my feet, and I don’t find my senior colleagues supportive. I’m only happy in the classroom with the learners, I’m less anxious in the classroom, but once I have to go to the staff room, I get anxious.”

(Participant 2)

Fieldnotes: There was a noticeable shift in participant 2’s posture when mentioning her time in the classroom; she was visibly relaxing and speaking with more confidence and comfort when discussing her interactions with learners. However, her demeanor changed dramatically when discussing her experiences in the staff room, exhibiting signs of anxiety, such as biting her lower lip and crossing her arms.

“I’m a loner at work, I spend my lunch time alone in my car, and on the days I do not have a car, I become miserable, but I sit in the classroom. In most times when I enter the staff room from my class, some of my colleagues will be talking, but when I enter, they keep quiet, and I could tell that they were talking about me, it is really hard going into such an environment on a daily basis.”

(Participant 6)

The above findings of this theme indicate that the mesosytem is supposed to create a healthy work environment, which will positively influence the participants’ experience of their microsystem. However, the participants’ mesosytem resulted in various aspects that created a hostile and isolating work environment, which the participants identified as work-related triggers of their mental illness relapse. This calls for targeted interventions to ensure that the mesosystem is conducive and accommodating of teachers with mental illness, to ensure they stay at work longer than being hospitalized due to mental illness relapse.

### 3.3. Theme 3: Exosystem

This theme yielded two subthemes. It explores how systemic and school-related pressures contribute to teachers’ mental health challenges and have the ability to trigger their mental illness relapse, and this is further explained in the subthemes below.

#### 3.3.1. Subtheme 3.1: Lack of Teacher Mental Health Considerations in Policies, High-Stakes Accountability Measures, and Systemic Policies Contribute to Reduced Well-Being

Participants mentioned that the school policies prioritize learners’ needs while neglecting teacher well-being, leaving them to manage their own mental health struggles without support. Furthermore, they struggle to meet policies’ daily expectations. Their experiences are further outlined below:

“Unfortunately, my mental health is not being catered for, all the school policies are focused on the leaners, they expect me as the teacher to be able to identify a distressed leaner, yet I am battling with my own mental health.”

(Participant 7)

“You know, the policy that says a leaner must not repeat a phase is a contributor to my poor mental health outcome because you find that a child is not able to read and write in grade 10 and this drops my pass rate and I am blamed for the poor pass rate, they do not consider that the leaner was condoned throughout, this hurts, really….and it makes me feel incompetent.”

(Participant 14)

Fieldnotes: Participant 14 showed visible frustration and discomfort while discussing the no-repeat policy, expressing feelings of incompetence due to being blamed for poor pass rates. Her tone was heavy, and she sighed multiple times.

#### 3.3.2. Subtheme 3.2: The Expectation of High Pass Rates for Learners by Parents and School Management, Without Providing Adequate Support to Teachers

Participants reported that they are expected to produce high pass rates, and they often do not receive support from parents and school management. Participants further mentioned that efforts to engage parents in their children’s education are frequently met with resistance or indifference. Participants’ experiences are further outlined below:

“Parents want their children to pass, but they do not meet us halfway, and when you ask them to come for a meeting in order to discuss their child’s performance, they make excuses. I always report to the HoD, and I’m known as the one who is always complaining and reporting incidents, but when I do not report, they blame me. At the end of each term, they won’t even consider the obstacles such as no parent involvement and all my efforts, I’m still blamed for learners not passing.”

(Participant 1)

“I get blamed as a teacher by the school management team for a low pass rate, but they don’t blame the other teachers who condoned those learners, and this frustrates me because if the child has been performing poorly throughout, what do they expect me to do?”

(Participant 8)

Fieldnotes: Participant 8’s tone was frustrated, and she appeared to be holding back feelings of resentment as she discussed the blame placed on her by the school management team. She shook her head in disbelief. Her body language suggested a mix of exhaustion and frustration, indicating that the situation was causing her significant emotional distress.

In the exosystem, the insufficient mental health policy considerations for teachers and the pressure to produce good results without sufficient support contribute to the participants’ emotional strain and serve as a trigger for their mental illness relapse. The exosystem highlights the need for broader institutional reforms that prioritize teacher well-being. This will positively impact the mesosystem and ultimately the microsystem.

## 4. Discussion

This study explored the work-related triggers of mental illness relapse among public school teachers in Limpopo Province, South Africa, where growing mental health needs intersect with educational and public health challenges. In alignment with Sustainable Development Goal 3 (good health and well-being) [25], the findings highlight a critical issue of teachers with existing mental illness in the workplace, in Sub-Saharan Africa, and this will be discussed using the theory lens [17].

At the microsystem level, the participants’ experiences show that excessive workload and administrative duties create major challenges and often trigger mental illness relapse. While a previous study conducted in France [26] confirms that heavy workloads negatively affect teachers’ mental well-being, it does not fully explain how this issue impacts teachers who already have mental illness. This study adds to the discussion by showing that, for teachers with mental illness, workload stress is not just an everyday challenge but a direct barrier to maintaining stability and increasing vulnerability to mental illness relapse and this was seen in the participants’ responses as they struggled to keep up with the excessive workload and the administrative duties which they perceive to be more than the teaching they have to do, and this affected their mental well-being and eventually triggered their mental illness relapse. Moreover, while Yean et al. found that administrative duties contribute to job dissatisfaction, they did not explore how this affects teachers with mental illness [27]. An important finding is that the participants often rely on outside help, such as teaching assistants or family members, to manage their workload. This suggests that their responsibilities are overwhelming and cannot be handled alone. Moreover, on the microsystem level, participants expressed classroom factors such as difficulty in maintaining order in their classrooms and learner behavior leading to teacher and learner altercations affecting their mental well-being. A South African study [28] also found that learner behavior, such as learner-to-teacher bullying, can result in negative emotions, low motivation, and disempowerment. For teachers with existing mental illness, such learner behavior not only hinders their ability to function optimally but can also serve as a trigger to mental illness relapse, as seen in the participants’ responses. These findings highlight how the microsystem (which the participants reported to be a high workload and administrative duties, classroom factors such as their interaction with learners) serves as a trigger for mental illness relapse, emphasizing the need for targeted interventions to ensure that their microsystem is conducive to their mental well-being.

At the mesosystem level, participants experienced being gossiped about by their colleagues, increased scrutiny and discrimination after disclosing their mental health condition, and a lack of support from their colleagues and the school management team, which not only affected their productivity but also served as a trigger to their mental illness relapse. Similarly, it was found that workplace gossip lowers psychological well-being, which is evident in participants as they suffered multiple relapses after becoming the subject of gossip [29]. Darr and Johns found that workers with mental illness who experience discrimination and gossip are more likely to suffer from stress, emotional strain, and depression [30]. This is reflected in participants’ experiences as they no longer enjoy going to work and feel that every action they take is unfairly linked to their diagnosis. These findings show that stigma in schools is not just harmful, it is actively worsening teachers’ mental health. This is particularly concerning as such experiences damage teachers’ confidence, sense of purpose, and hope for the future [31]. Schools must recognize that ignoring workplace stigma is not just a failure of leadership, it is a direct threat to both teacher well-being and the quality of education. Furthermore, when individuals feel excluded, unsupported, or fearful of seeking help, the resultant anxiety and distress can severely impair their ability to perform effectively in their roles [32]. This is evident in the participants’ experiences of a lack of support from senior staff members and their fear of asking for assistance due to the unsupportive work environment. These personal accounts highlight the toxic nature of workplaces that neglect mental health support and demonstrate how the mesosystem level can be a trigger for mental illness relapse.

At the exosystem level, participants reported work aspects such as the lack of teacher mental health considerations in policies, systemic policies, and expectations of high pass rates for learners by parents and school management as a trigger for their mental illness relapse. Novotney emphasizes the importance of integrating mental health into workplace policies to prevent psychological distress, enhance worker connection, and validate their well-being [33]. While the Occupational Health and Safety Act 85 outlines employers’ responsibilities to provide a safe working environment, including mental health support services through programs like the Employee Assistance Program (EAP), the effectiveness of such policies in schools appears limited [34]. The EAP is structured as a referral system, which may not be immediately accessible in school settings, creating a significant gap in the provision of timely mental health support for teachers. Furthermore, the unrealistic expectation of high pass rates, along with insufficient support mechanisms, poses a significant threat to the mental well-being of teachers and serves as a trigger for mental illness relapse. Jerrim and Sims highlight that results-oriented systems, prevalent in many countries, contribute to heightened stress among educators, with teachers bearing the burden of accountability for student outcomes [35]. While this is an important observation, it fails to acknowledge that such systems often prioritize results over the necessary support structures that should enable teachers to succeed. In this context, the study also draws on various findings [36,37] to underscore the pressure faced by teachers in high-profile schools or those with poor student performance. While these studies highlight the pressures placed on educators, they fail to critically address the role that the exosystem plays in relation to educational policies and practices, such as grade condonation and inadequate learner support, in perpetuating the cycle of blame, which has the potential for affecting teachers with existing mental illness and has the ability to trigger their mental illness relapse.

## 5. Conclusions

Bronfenbrenner’s Ecological Systems Theory [17] indicated that there are various interconnected work factors that have the potential to trigger mental illness relapse and this was seen in the microsystem (including classroom factors such as teachers struggling to control learners and also gossip and an excessive workload), the mesosystem (this was seen by the poor support the teachers received at work, being gossiped about by their colleagues, increased scrutiny and stigma after disclosing their mental health conditions at work, thus affecting their productivity and ability to seek assistance when required), and the exosystem (this was seen by the lack of adequate school-based policies that address teacher mental health and the expectations of high pass rate for learners by parents and the school management).

Various studies shows that, although teachers without mental illness also experience work stress as per other studies, they are still able to function in the midst of their stress, however, this study highlights that, for teachers with existing mental illness, it triggers their mental illness relapse, causing them to regress and the danger in this may consequently result in hospitalization as seen in the participants’ demographic information that they all had multiple relapses. This then emphasizes that they spend a lot of time away from work. If the triggers are not dealt with, this will consequently lead to treatment resistance and cognitive impairment due to progressive structural brain damage [4], thus affecting their health and well-being.

Moreover, the findings of this study demonstrate several consistent work-related triggers of mental illness relapse across all school settings. However, there were also patterns related to the career stage. Within the mesosystem, early-career and late-career teachers described distinct relational challenges with colleagues that contributed to their mental distress. This was seen when an early-career teacher (participant 2, age 26) reported feelings of anxiety and isolation due to perceived lack of support from senior colleagues. Similarly, a late-career teacher (participant 6, age 50) described social exclusion and mistrust from colleagues, which also contributed to feelings of distress. These accounts suggest that, while all participants may experience psychosocial stressors resulting from their mesosystem, the specific nature and triggers of those stressors may vary depending on one’s career stage.

The study therefore recommends collaboration between various stakeholders such as occupational therapists, psychologists, psychiatrists, policy makers, the Department of Basic Education, union representatives, and the academic community to critically look at the raised work-related triggers of mental illness and come up with a solution on how best they can make the work environment a conducive and healthy one for teachers with mental illness.

From the participants’ responses, it is crucial to strengthen the exosystem by developing school-based policies that will mitigate issues in the mesosystem, such as work gossip, workplace exclusion, and lack of support by colleagues. This will ultimately enable the participants to manage and be able to cope with the demands of the microsystem, such as excessive workload and classroom factors. This is significant because the absence of comprehensive school-based mental health policies, as highlighted by participants, emphasizes the inadequacy of the existing structures in addressing the unique challenges faced by teachers with mental illness. As the World Health Organization (WHO) reports, the failure to incorporate mental health into workplace policies contributes to absenteeism and higher rates of mental illness relapse [38]. By addressing the work-related triggers of mental illness relapse, this study contributes to enhancing teacher well-being, retention, and effectiveness, which are essential for achieving quality education and fostering a healthy, productive workforce in line with the United Nations Sustainable Development Goal 3, which will eventually positively impact on Goal 4 of quality education [25].

## 6. Limitations of the Study

This study was only conducted on public school teachers. Therefore, future research can compare public schools and private schools for a more holistic consideration of environmental context dynamics. The study used qualitative methodology; it only focused on Limpopo Province and was conducted in some parts of the province, which may influence how work-related triggers of mental illness are experienced and interpreted. Although the sample size of 14 participants was determined by data saturation, this is a further limitation of the study. While the study provides rich data on the teachers’ lived experiences of work-related triggers of their mental illness relapse, the sample size may limit the transferability of the findings to other provinces in South Africa or various countries. Future studies involving larger and more varied samples may help to further validate and expand upon the findings of this study. Moreover, Bronfenbrenner’s Ecological Systems Theory [17] encompasses five interrelated systems; this study only focused on the microsystem, mesosystem, and exosystem, as these levels are most relevant to exploring work-related triggers of mental illness relapse. The macrosystem and chronosystem were beyond the scope of this study, as the data did not include broader socio-cultural or longitudinal factors. Future research could expand on this by exploring how cultural stigma, national policies, and long-term professional factors trigger mental illness relapses over time.

## Figures and Tables

**Table 1:** Participants’ demographic information.

Participant Number	Age	Sex	Diagnosis	Number of Relapses	School Level	Current Status
1	38	Female	Bipolar disorder	5	Primary	Long-term incapacity leave
2	26	Female	Major depressive disorder	3	High	Back at work
3	46	Female	Major depressive disorder	4	High	Long-term incapacity leave
4	35	Female	Major depressive disorder	3	High	Back at work
5	37	Female	Bipolar disorder	4	Primary	Long-term incapacity leave
6	50	Female	Schizophrenia	6	Primary	Admitted
7	38	Male	Major depressive disorder	4	Primary	Back at work
8	45	Female	Bipolar disorder	5	High	Admitted
9	40	Male	Schizophrenia	4	High	Long-term incapacity leave
10	39	Male	Major depressive disorder	6	Primary	Back at work
11	42	Female	Major depressive disorder	5	Primary	Back at work
12	36	Male	Generalized anxiety disorder	3	High	Long-term incapacity leave
13	50	Male	Schizophrenia	4	High	Long-term incapacity leave
14	53	Male	Bipolar disorder	6	High	Admitted

**Table 2:** Themes and subthemes.

Themes	Subthemes
Microsystem	1.1: Excessive workload and administrative responsibilities
	1.2: Classroom factors
Mesosystem	2.1: Gossip in the workplace
	2.2. Disclosure of mental health conditions at the workplace leads to decreased productivity
	2.3. Workplace exclusion and lack of support from colleagues
Exosystem	3.1: Lack of teacher mental health considerations in policies, high-stakes accountability measures, and systemic policies contribute to reduced well-being
	3.2: Expectations of high pass rates for learners by parents and school management

## Data Availability

Due to the sensitivity of the data collected, the data generated or analyzed during the current study is not publicly available but can be requested from Nkomo.
